# Prognostic implications of preoperative systemic inflammatory markers in oral squamous cell carcinoma, and correlations with the local immune tumor microenvironment

**DOI:** 10.3389/fimmu.2022.941351

**Published:** 2022-07-26

**Authors:** Marta Ruiz-Ranz, Paloma Lequerica-Fernández, Tania Rodríguez-Santamarta, Faustino J. Suárez-Sánchez, Rosa M. López-Pintor, Juana M. García-Pedrero, Juan C. de Vicente

**Affiliations:** ^1^ Department of Oral and Maxillofacial Surgery, Hospital Universitario Central de Asturias (HUCA), Oviedo, Spain; ^2^ Department of Biochemistry, Hospital Universitario Central de Asturias (HUCA), Oviedo, Spain; ^3^ Instituto de Investigación Sanitaria del Principado de Asturias (ISPA), Instituto Universitario de Oncología del Principado de Asturias (IUOPA), Universidad de Oviedo, Oviedo, Spain; ^4^ Department of Pathology. Hospital Universitario de Cabueñes, Gijón, Spain; ^5^ ORALMED Research Group, Department of Dental Clinical Specialties, School of Dentistry, Complutense University, Madrid, Spain; ^6^ Department of Otolaryngology, Hospital Universitario Central de Asturias (HUCA), Oviedo, Spain; ^7^ Centro de Investigación Biomédica en Red de Cáncer (CIBERONC), Instituto de Salud Carlos III, Madrid, Spain; ^8^ Department of Surgery, University of Oviedo, Oviedo, Spain

**Keywords:** LMR, NLR, oral cancer, PLR, prognosis, systemic immune-inflammation index, tumor microenvironment

## Abstract

**Purpose:**

The aim of this study was to investigate the prognostic significance of preoperative inflammatory markers in peripheral blood of patients with oral squamous cell carcinoma (OSCC), and to establish correlations with the infiltrate of macrophages and lymphocytes in the local immune tumor microenvironment (TME).

**Materials and Methods:**

Neutrophil-lymphocyte ratio (NLR), platelet-lymphocyte ratio (PLR), lymphocyte-monocyte ratio (LMR), and systemic immune-inflammation index (SII) were retrospectively evaluated in a cohort of 348 OSCC patients, and correlated with overall (OS) and disease-specific survival (DSS). Immunohistochemical analysis of tumoral and stromal infiltration of CD8+, CD4+, FOXP3+ and CD20+ lymphocytes and CD68+ and CD163+ macrophages was performed in a subset of 119 OSCC patient samples, and correlations further assessed.

**Results:**

NLR, SII, and LMR were significantly associated with a poorer OS in univariate analysis; however, only NLR remained a significant independent predictor in the multivariate analysis (HR = 1.626, *p* = 0.04). NLR and SII were inversely and significantly correlated with stromal infiltration of CD8+, CD4+, and CD20+ lymphocytes. Moreover, a significant correlation between LMR was also found to significantly associate with stromal infiltration of CD8+, CD4+, and CD20+ lymphocytes, stromal CD68+ and CD163+ macrophages, and also tumoral infiltration of CD4+ and CD20+ lymphocytes.

**Conclusions:**

Preoperative NLR, SII, and LMR may serve as valuable systemic markers to predict OSCC patient survival, with NLR emerging as an independent predictor of poor OS. Moreover, strong significant correlations were exclusively observed between systemic inflammatory markers and the local stromal infiltration of lymphocytes in the TME.

## Introduction

Oral squamous cell carcinoma (OSCC) is one of the most common tumors in the world, with a global incidence of 377,713 new cases in 2020 (1). Due to the lack of specific symptoms in the early stage of this entity, most OSCC patients are in an advanced stage at the time of diagnosis, more than half of them suffer from a recurrence within 2 years of the initial surgical treatment (2), and the 5-year survival remains approximately 60% (3). Tumor node metastasis (TNM) stage has been established as the main determinant of prognosis and treatment planning for OSCC (3). However, this factor is far from optimal in predictive accuracy, because patients with the same TNM stages often exhibit significant differences in prognosis (4, 5).

Chronic inflammation was firstly linked to carcinogenesis by Rudolf Virchow in 1863 (6). Nowadays, it is recognized as one of the hallmarks of cancer (7), since inflammation induces angiogenesis, cell proliferation, DNA damage caused by reactive oxygen species, and inhibits apoptosis of cancer cells (8). In the tumor microenvironment (TME), tumor cells reprogram surrounding stromal cells, leading to an inflammatory response with expansion and recruitment of different immune cells to support cancer progression (9). Moreover, it has been hypothesized that local inflammatory conditions lead to a systemic inflammatory state that could be measured through peripheral blood markers. In fact, various systemic markers have been correlated with worse clinical outcomes in a broad spectrum of cancers, including head and neck squamous cell carcinoma (HNSCC), colorectal, non-small cell lung, stomach, esophageal, liver, gallbladder, and prostate cancers, among others (10–17).The magnitude of the inflammatory response can be thoroughly explored by malnutrition and several indicators, among which the counts of neutrophils, lymphocytes, monocytes, and platelets in peripheral blood are readily available. Moreover, systemic ratios between the aforementioned cells, such as neutrophil-lymphocyte ratio (NLR), platelet-lymphocyte ratio (PLR), and lymphocyte-monocyte ratio (LMR) have been shown to correlate with survival in cancer patients (8, 10, 12, 18). Recently, the systemic immune-inflammation index (SII) based on neutrophils, lymphocytes and platelets has been proposed as a joint tool to offer helpful prognostic information in patients with hepatocellular carcinoma, pancreatic cancer and germ-cell tumor (19–21), and it has also been tested in OSCC (22). Despite the studies published, the prognostic value of NLR, PLR, LMR, and SII in patients with HNSCC and in OSCC based on multivariate analysis remains unclear, or even controversial (11, 14, 23). Inconsistent findings may be attributed to differences in sample sizes, tumor sites and progression, cutoff values used among the studies, which require further verification. Noteworthy, these markers are rarely studied together in the same patient series, and the relationships between cell numbers and ratios in blood and those locally detected in tumor samples for the intrinsic TME have not yet been properly assessed in OSCC.

The aim of this study was to compare the prognostic performance of preoperative NLR, PLR, MLR, and SII in a large homogeneous cohort of 348 patients with OSCC treated with radical surgery. In addition, we further assessed the correlations between these systemic inflammatory markers in peripheral blood with the stromal and tumoral infiltrate of macrophages, T and B lymphocytes locally detected by immunohistochemistry in OSCC tissue samples.

## Materials and methods

### Patients

The present retrospective study was performed in accordance with the Declaration of Helsinki and following approval by the Regional Ethics Committee from Principado de Asturias (date of approval 14th of May 2019; approval number: 136/19, for the project PI19/01255), encompassing all cases of patients with OSCC treated by surgery between January 1996 and November 2007 at the Hospital Universitario Central de Asturias, Spain. Inclusion criteria were defined as follows: (1) histologically confirmed OSCC, (2) no previous history of other head and neck cancer, (3) no radiotherapy and/or chemotherapy treatment prior to surgery, (4) a minimum follow-up time of 3 years in the censored cases, and (5) availability of complete clinical, pathological and laboratory information. Exclusion criteria include inflammatory conditions, corticosteroid therapy, and treatment with platelet aggregation inhibitors.

A total of 348 OSCC patients were included in the study, and their clinicopathologic data were collected from clinical records. Data related to laboratory tests were extracted from electronic medical records. Clinical and pathological characteristics included age, gender, body mass index (BMI), smoking and or drinking habits, tumor, neck node and clinical stages, histological grade of differentiation, and tumor location. Patients were staged according to the 8^th^ edition of the TNM classification of malignant tumors (24).

### Determination of blood counts

Complete blood count was preoperatively ordered 1 week before the treatment. Neutrophil, lymphocyte, monocyte and platelet count were obtained. NLR was calculated by dividing the absolute neutrophil (N) count by the absolute lymphocyte (L) count (NLR = N/L). PLR was calculated by dividing the absolute platelet (P) count by the absolute lymphocyte count (PLR = P/L). LMR by dividing the absolute lymphocyte (L) count by the absolute monocyte (M) count as determined from the complete blood cell count (LMR = L/M). The SII index was calculated by multiplying the absolute platelet (P) and neutrophil (N) counts and then dividing this product by the absolute lymphocyte (L) count (SII = P x N/L). Accordingly, SII can also be calculated by multiplying the absolute platelet (P) count and the NLR.

### Immunohistochemical analysis in OSCC tissue specimens

Immunohistochemical evaluation was performed in a subset of 119 patients an in order to determine the densities of tumor-infiltrating T- and B-cell lymphocytes and macrophages in the OSCC TME, thereby separately evaluating different immune cell populations (CD4+, CD8+, FOXP3+, CD20+, CD68+ and CD163+) in the tumor nests and also the surrounding tumor stroma. The immunohistochemical (IHC) analysis was carried out using OSCC tissue microarrays (TMAs), as previously described (25). The TMAs were cut into 3 μm sections and dried on Flex IHC microscope slides (DakoCytomation, Glostrup, Denmark). The sections were deparaffinized in xylene and rehydrated through a graded alcohol series. Antigen retrieval was performed by heating the sections with Envision Flex Target Retrieval solution, high pH (Dako, Glostrup, Denmark). Staining was done at room temperature on an automatic staining workstation (Dako Autostainer Plus, Dako). The following primary antibodies were used: mouse monoclonal anti-CD4 (Dako, clone 4B12, 1:80 dilution), mouse monoclonal anti-CD8 (Dako, clone C8/144B, prediluted), rabbit monoclonal anti-FoxP3 (Cell Signalling Technology, clone D6O8R, 1:100 dilution), mouse monoclonal anti-CD20 (Dako, clone L26, catalogue number M0755; 1:200 dilution), anti-CD68 (Agilent-Dako, clone KP1, prediluted), and anti-CD163 (Biocare Medical, Pacheco, CA, USA; clone 10D6, 1:100 dilution), by using the Dako EnVision Flex + Visualization System (Dako Autostainer) and diaminobenzidine chromogen as substrate. Negative controls were prepared by omitting the primary antibody. Positive controls were prepared using appropriate positive control slides. Counterstaining with hematoxylin was the final step. CD4, CD8, FOXP3, CD20, CD68, and CD163 immunostainings in both the tumor nests and the surrounding stroma were scored using the average of positively stained cells in each 1 mm^2^ area from three independent high-power representative microscopic fields (HPFs, 400 x; 0.0625 μm^2^).

### Statistical analysis

Continuous normally distributed variables were presented as mean and standard deviation, and the categorical data were expressed as percentage. Spearman correlation coefficient was used to analyze the correlation between continuous variables, and the non-parametric Mann-Whitney and Kruskal-Wallis tests were used to study the associations between the continuous variables NLR, PLR, SII, LMR with the subgroups of patients. Receiver operating curve (ROC) analysis was applied to determine ideal cutoff values of NLR, PLR, LMR and SII. Survival curves were performed by Kaplan-Meier analysis and log-rank test. Univariate and multivariate Cox proportional hazard regression model was also performed. Hazard ratios (HR) with 95% confidence intervals were calculated. The endpoints of the survival analysis were overall survival (OS) and disease-specific survival (DSS). OS was measured as the time interval from the initial treatment to the time of death from any cause or the last date of observation, and DSS as the time interval from the initial treatment to the date of death caused by disease progression. *p* values less than 0.05 was considered statistically significant. The statistical analysis was undertaken using SPSS version 21.0 (IBM Co., Armonk, NY, USA).

## Results

### Patient characteristics

Detailed demographic and clinicopathological characteristics of 348 patients are shown in **Table 1**. In short, patients were predominantly men (63.5%) and ranged in age from 28 to 92 years (mean/median 62 years). Smoking habit and alcohol consumption were respectively reported in 58% and 46% of patients. The most common primary site was the tongue (n = 144, 41.4%), followed by the floor of the mouth (n = 77, 22.1%). Clinical stages were as follows: stage I (n = 75, 21.6%); stage II (n = 98, 28.2%); stage III (n = 62, 17.8%); and stage IV (n = 113, 32.5%). A majority of the enrolled patients had pN0 disease (n = 211, 63.2%), and well-differentiated tumors (n = 216, 62%). All patients underwent surgery of the primary tumor with curative intention, and none of them received preoperative treatment with radiotherapy and/or chemotherapy. A total of 174 (50%) patients received adjuvant radiotherapy, and 35 (10%) of them, received also chemotherapy.

**Table 1:** Clinical and pathological characteristics of the cohort of 348 OSCC patients selected for study.

Variable	Number (%)
Age (year) (mean ± SD; median; range) <65 years >65 years	62.36 ± 13.19; 62; 28 – 92 192 (55) 156 (45)
BMI (kg/m^2^) (mean ± SD; median; range)	26.34 ± 4.68; 26.34; 16.90 – 51.75
Gender	
Men Women	221 (63.5) 127 (36.5)
Tobacco use	
Smoker Non-smoker Unknown	202 (58) 127 (36.5) 19 (5.5)
Alcohol use	
Drinker Non-drinker Unknown	160 (46) 172 (49.4) 16 (4.6)
Location of primary tumor	
Tongue Floor of the mouth Gingiva Palate Buccal cheek mucosa Retromolar trigone Multicenter	144 (41.4) 77 (22.1) 61 (17.5) 18 (5.2) 24 (6.9) 22 (6.3) 2 (0.6)
AJCC pT	
pT1 pT2 pT3 pT4	89 (26.2) 142 (41.8) 49 (14.4) 60 (17.6)
AJCC pN	
pN0 pN1 pN2 pN3	211 (63.2) 57 (17.1) 64 (19.2) 2 (0.6)
AJCC	
I II III IV	75 (21.6) 98 (28.2) 62 (17.8) 113 (32.5)
G status	
G1 G2 G3	216 (62.1) 118 (33.9) 14 (4.0)
Clinical status at the end of the follow-up	
Alive and without recurrence Dead of index cancer Lost or died of other causes (censored) Second primary tumor	143 (41.1) 140 (40.2) 61 (17.5) 4 (1.1)

### Determination of NLR, PLR, SII, LMR and associations with clinicopathologic characteristics of OSCC patients

Differential white blood cell and platelet counts were preoperatively determined in peripheral blood from 348 OSCC patients, and the ratios of NLR, PLR, SII, LMR were calculated and summarized in **Table 2**. High levels of NLR and SII were robustly and significantly associated with larger tumor size (*p* = 0.001, *p* < 0.0001, respectively) and higher American Joint Committee on Cancer (AJCC) stage (*p* = 0.009, *p* = 0.003, respectively) (**Table 3**). Moreover, mean values for NLR and SII gradually increased from early to late clinical stages. There was a trend to association between high mean values of NLR, PLR, SII and LMR, and the presence of lymph neck node metastasis, although none reached statistical significance. No significant associations between NLR, PLR, SII, LMR and other clinicopathological variables (age, sex, tobacco or alcohol consumption, histological grade of differentiation and clinical status at the end of the follow-up) were observed, with the only exceptions of SII and alcohol consumption (*p* = 0.039), and PLR and LMR with tumor location (*p* = 0.002). All four studied inflammatory ratios showed an inverse correlation with the body mass index; however, only led to significant differences in the case of SII and PLR (**Table 3**).

**Table 2:** Evaluation of inflammatory markers and ratios in peripheral blood from 348 OSCC patients.

Parameter	Mean ± SD, median (range)
**White blood cell count**	
Neutrophil count, 10^9^/L	4.72 ± 1.78, 4.30 (1.10 – 10.70)
Lymphocyte count, 10^9^/L	2.15 ± 0.97, 2.00 (0.40 – 11.70)
Platelet count, 10^9^/L	254.97 ± 79.24; 249; 67 - 659
Monocyte count, 10^9^/L	0.65 ± 0.70, 0.55 (0.00 – 9.00)
**Calculated ratios**	
NLR	2.48 ± 1.26; 2.22 (0.25 – 7.86)
PLR	135.35 ± 58.35, 123.84 (18.03 – 416.25)
SII	639.87 ± 408.57, 524.85 (38.75 – 2,634.05)
LMR	3.96 ± 2.45 (0.255 – 27.00)

NLR, neutrophil-lymphocyte ratio; SII, systemic immune-inflammation index; MLR, monocyte-lymphocyte ratio; PLR, platelet lymphocyte ratio.

**Table 3:** Relationships between the clinicopathological variables and inflammatory ratios calculated for the cohort of 348 OSCC patients.

Variable	Total No.	NLR Mean (SD)	*p*	SII Mean (SD)	*p*	LMR Mean (SD)	*p*	PLR Mean (SD)	*p*
**BMI**	321	-0.114 *	0.054	-0.140 *	0.019	0.11 *	0.063	-0.131 *	0.027
**Age (years)**									
** < 65** ** ≥ 65**	177 135	2.57 (1.80) 2.49 (1.18)	0.65	666.53 (455.23) 604.98 (336.44)	0.17	4.04 (2.76) 3.87 (1.99)	0.53	132.51 (59.84) 139.18 (56.23)	0.08
**Gender**									
** Female** ** Male**	116 196	2.46 (1.15) 2.59 (1.76)	0.44	658.49 (373.57) 628.87 (428.46)	0.54	4.15 (1.71) 3.85 (2.81)	0.30	143.53 (55.88) 130.03 (59.14)	0.31
**Tobacco**									
** No** ** Yes**	112 181	2.47 (1.11) 2.59 (1.80)	0.48	615.55 (320.71) 655.80 (442.30)	0.37	3.95 (1.97) 4.01 (2.79)	0.84	140.20 (56.00) 132.03 (59.41)	0.81
**Alcohol consumption**									
** No** ** Yes**	155 141	2.42 (1.19) 2.62 (1.86)	0.25	584.77 (317.14) 681.55 (459.21)	0.039	4.01 (2.04) 4.01 (2.93)	0.99	134.31 (53.08) 136.08 (62.90)	0.80
**Location of primary tumor**									
** Tongue** ** Floor of the mouth** ** Gingiva** ** Palate** ** Buccal cheek mucosa** ** Retromolar trigone** ** Multicenter**	144 77 61 18 24 22 2	2.58 (1.87) 2.37 (1.26) 2.57 (1.32) 2.83 (1.53) 2.59 (1.48) 2.58 (1.00) 1.44 (1.68)	0.87	645.92 (409.32) 601.63 (404.32) 709.17 (484.22) 649.75 (458.42) 584.10 (266.51) 621.37 (263.82) 336.89 (421.64)	0.72	3.77 (1.87) 4.47 (3.55) 3.79 (2.38) 3.54 (1.38) 4.32 (1.87) 3.39 (1.25) 10.59 (5.75)	0.002	140.17 (63.14) 121.86 (53.93) 142.61 (50.44) 139.87 (62.29) 134.55 (65.71) 134.33 (44.36) 0	0.001
**AJCC pT**									
** pT1** ** pT2** ** pT3** ** pT4**	80 131 42 52	2.09 (1.02) 2.44 (1.24) 2.97 (1.14) 3.05 (2.67)	0.001	536.59 (346.73) 579.87 (357.65) 841.22 (401.80) 744.63 (495.79)	< 0.0005	4.49 (2.36) 3.91 (2.01) 3.27 (1.11) 4.03 (3.96)	0.06	123.82 (50.84) 126.07 (52.60) 165.73 (51.28) 152.20 (76.96)	0.24
**AJCC pN**									
** pN0** ** pN+**	193 107	2.46 (1.22) 2.62 (2.00)	0.37	628.05 (384.10) 639.94 (398.37)	0.80	3.94 (2.07) 4.10 (3.12)	0.58	138.47 (59.07) 129.02 (56.24)	0.10
**Stage**									
** I** ** II** ** III** ** IV**	70 89 52 101	2.03 (1.04) 2.52 (1.23) 2.74 (1.23) 2.81 (2.11)	0.009	516.28 (352.15) 598.56 (328.90) 694.15 (371.66) 738.41 (497.74)	0.003	4.62 (2.76) 3.84 (1.85) 3.53 (1.45) 3.84 (3.00)	0.06	122.79 (54.21) 132.63 (48.28) 135.22 (50.52) 148.16 (71.55)	0.37
**Postoperative radiotherapy**									
** No** ** Yes**	153 151	2.51 (1.33) 2.53 (1.76)	0.57	626.10 (425.25) 647.07 (381.10)	0.55	3.84 (2.11) 4.16 (2.80)	0.37	136.39 (59.47) 127.97 (52.79)	0.32
**Grade**									
** Well** ** Moderate + Poor**	191 121	2.44 (1.29) 2.69 (1.91)	0.17	629.15 (425.72) 656.80 (381.03)	0.56	4.08 (2.80) 3.77 (1.77)	0.27	132.71 (58.41) 139.78 (57.77)	0.45
**Clinical status at the end of the follow-up**									
** Alive and without recurrence** ** Dead of index cancer** ** Lost or died of other causes** ** Second primary tumor**	130 120 58 4	2.44 (1.15) 2.66 (1.93) 2.56 (1.53) 2.27 (1.68)	0.71	602.56 (326.49) 676.69 (457.15) 636.85 (447.01) 800.10 (699.74)	0.45	4.03 (2.07) 3.98 (2.98) 3.75 (2.07) 4.52 (2.09)	0.86	132.36 (54.21) 134.65 (48.28) 144.11 (50.52) 152.74 (72.79)	0.13

BMI, Body mass index. * Pearson correlation coefficient.

### Determination of cutoff values for NLR, PLR, SII, LMR and associations with patient prognosis

Patients were followed up for 280 months (median 54 months). The median DSS and OS times were 141 months (range 108 to 173 months) and 70 months (range 48 to 91 months), respectively. The 1-year, 3-year and 5-year survival rates were 78%, 63% and 54%, respectively. The areas under curve (AUC) for NLR, PLR, SII and LMR were 0.520, 0.519, 0.532 and 0.447, respectively. Receiver operating curve (ROC) analyses were performed for all calculated ratios, but significant cutoff values based on the ROC analyses failed to show any prognostic relevance. Thus, in order to find any relevant prognostic significance of these ratios, the cohort was divided according to the different percentiles of cell ratios. When the percentile 90 was used as cutoff value a significant association with OS in NLR and SII (4.08 and 1,137, respectively) was observed, and the same was found with the percentile 75 of LMR (4.58) (**Table 4**).

**Table 4:** Univariate Cox regression analysis of overall and disease-specific survival in the cohort of 348 OSCC patients.

Variable	Overall survival		Disease-specific survival	
	HR (95% CI)	*p*	HR (95% CI)	*p*
**Age (years)**				
** <65** ** ≥65**	1 (reference) 1.439 (1.086 – 1.908)	0.01	1 (reference) 1.325 (0.948 – 1.852)	0.10
**Gender**				
** Female** ** Male**	1 (reference) 1.316 (0.978 – 1.771)	0.06	1 (reference) 1.140 (0.805 – 1.614)	0.46
**Tobacco**				
** No** ** Yes**	1 (reference) 1.099 (0.854 – 1.413)	0.60	1 (reference) 1.046 (0.770 – 1.413)	0.77
**Alcohol**				
** No** ** Yes**	1 (reference) 1.222 (0.958 – 1.558)	0.07	1 (reference) 1.075 (0.800 – 1.430)	0.62
**AJCC pT**				
** pT1** ** pT2** ** pT3** ** pT4**	1 (reference) 1.383 (0.936 – 2.045) 2.027 (1.250 – 3.288) 3.519 (2.280 – 5.430)	<0.0001 0.10 0.004 <0.0001	1 (reference) 1.362 (0.835 – 2.220) 2.202 (1.223 – 3.963) 4.477 (2.691 – 7.446)	<0.0001 0.21 0.008 <0.0001
**AJCC pN**				
** pN0** ** pN+**	1 (reference) 2.583 (1.938 – 3.442)	<0.0001	1 (reference) 3.158 (2.236 – 4.461)	<0.0001
**Stage**				
** I** ** II** ** III** ** IV**	1 (reference) 1.377 (0.862 – 2.201) 1.967 (1.203 – 3.215) 3.603 (2.349 – 5.526)	<0.0001 0.18 0.007 <0.0001	1 (reference) 1.594 (0.854 – 2.975) 1.967 (1.431 – 5.059) 5.069 (2.893 – 8.881)	<0.0001 0.14 0.002 <0.0001
**Postoperative radiotherapy**				
** No** ** Yes**	1 (reference) 1.422 (1.228 – 1.647)	<0.0001	1 (reference) 1.572 (1.336 – 1.849)	<0.0001
**Grade**				
** Well** ** Moderate + Poor**	1 (reference) 1.216 (0.915 – 1.616)	0.17	1 (reference) 1.315 (0.940 – 1.841)	0.11
**NLR**				
** ≤4.08** ** >4.08**	1 (reference) 1.847 (1.179 – 2.895)	0.007	1 (reference) 1.644 (0.956 – 2.829)	0.07
**PLR**				
** ≤205** ** >205**	1 (reference) 1.465 (0.908 – 2.363)	0.11	1 (reference) 1.617 (0.939 – 2.784)	0.08
**SII**				
** ≤1,137** ** >1,137**	1 (reference) 1.979 (1.260 – 3.108)	0.003	1 (reference) 1.479 (0.829 – 2.638)	0.18
**LMR**				
** ≤4.58** ** >4.58**	1 (reference) 0.668 (0.466 – 0.957)	0.02	1 (reference) 0.665 (0.426 – 1.036)	0.07

Consequently, patients were divided into two groups based on these cutoff points (low vs high values). As summarized in **Table 4**, results from univariate Cox analysis show that age, T, N, clinical stage, postoperative radiotherapy, NLR, SII, and LMR, but not PLR, were significantly associated with OS. Even though all four inflammatory ratios showed trend to association with DSS, T, N, stage, and postoperative radiotherapy were the only parameters significantly associated with DSS. Mean and median OS rates were consistently and significantly longer in patients with low NLR and SII values (below their respective cutoff values of 4.08 and 1,137; HR = 1.847 and 1.979, respectively), while longer OS was found in patients with high LMR value (above its respective cutoff value of 4.58; HR = 0.668). A multivariate Cox regression analysis was performed using those variables that were statistically significant from univariate statistical analyses. This analysis showed that age higher than 65 years (HR = 1.498, *p* = 0.003), clinical stages III (HR = 2.140, *p* = 0.005) and IV (HR = 3.708, *p* = < 0.0001), postoperative radiotherapy (HR = 1.431, *p* = < 0.0001) and NLR higher than 4.08 (HR = 1.626, *p* = 0.04) were independent predictors of a worse OS. Only clinical stages III and IV, and postoperative radiotherapy retained a significant independent association with DSS in the multivariate analysis (**Table 5**). Finally, we performed a subgroup analysis of the oncological endpoints in postoperatively irradiated patients only, due to this could be a more homogeneous cohort in terms of stage and risk factors, and response to irradiation strongly depends on the immune system. Results from this new analysis are now provided as **Supplementary Information Table S1**. As a brief summary of these new data, only age and the tumor size were significantly associated with OS and DSS in the subgroup of irradiated OSCC patients. Even though there was a trend between high NLR and SII and a poorer OS, differences did not reach statistical significance. Moreover, major poor prognostic factors such as pN classification failed not show significant associations with OS and DSS for this subset of patients, probably because the effect of these clinical variables was lost by specifically selecting for analysis only the subgroup of irradiated patients.

**Table 5:** Multivariate Cox regression analysis of overall and disease-specific survival in the cohort of 348 OSCC patients.

Variables	Overall survival		Disease-specific survival	
	HR (95% CI)	*p*	HR (95% CI)	*p*
Age				
≤65 years	1 (reference)	0.017		
>65 years	1.498 (1.095 – 2.051)			
Stage		< 0.0001		< 0.0001
I	1 (reference)		1 (reference)	
II	1.459 (0.956 – 2.227)	0.08	1.470 (0.855 – 2.526)	0.16
III	2.140 (1.255 – 3.649)	0.005	2.690 (1.399 – 5.170)	0.003
IV	3.708 (2.290 – 6.002)	<0.0001	5.112 (2.873 – 9.095)	<0.0001
Radiotherapy				
No	1 (reference)	< 0.0001	1 (reference)	< 0.0001
Yes	1.431 (1.212– 1.689)		1.598 (1.313 – 1.945)	
NLR				
≤4.08	1 (reference)	0.04		
>4.08	1.626 (1.004 – 2.633)			

### Correlation analysis between systemic NLR, PLR, SII, LMR, and the tumor immune infiltrate in OSCC tissue samples

We next evaluated by immunohistochemistry the densities of tumor-infiltrating T- and B-cell lymphocytes and macrophages in the OSCC TME in a subset of 119 patients, to correlate the IHC data from OSCC tissue samples with the systemic inflammatory ratios determined in peripheral blood from the same subset of OSCC patients. Thus, density numbers of infiltrating CD8+, CD4+, and FOXP3+ T-cell lymphocytes, CD20+ T-cell lymphocytes, and CD68+ and CD163+ macrophages were separately evaluated in both the tumor nests and surrounding stroma, and the mean numbers were correlated with the mean values for NLR, PLR, SII and LMR (**Table 6**). We found inverse significant correlations between NLR and SII with stromal CD8+, CD4+, and CD20+ infiltrating lymphocytes. In addition, positive significant correlations were observed between LMR and stromal infiltration of CD8+, CD4+, CD20+ lymphocytes, stromal infiltration of CD68+ and CD163+ macrophages, as well as tumoral CD4+ and CD20+ infiltrating lymphocytes (**Table 6**). PLR did not show any significant correlation with the immune tumor infiltrate, nor FOXP3+ TILs were correlated with any of the systemic inflammatory ratios (**Table 6**). Noteworthy, strong significant correlations between systemic inflammatory markers and the immune cell infiltrate in the OSCC TME were exclusively observed with immune cells infiltrating the tumor stroma, but not the tumor nests.

**Table 6:** Correlations between systemic NLR, SII, PLR, and LMR, and the immune tumor infiltrate in OSCC tissue samples from 119 patients.

Variable	NLR	SII	PLR	LMR
Stromal CD8+ TILs	-0.318 **< 0.001**	-0.26 **< 0.001**	-0.170 0.09	0.442 **< 0.0001**
Tumoral CD8+ TILs	-0.132 0.18	-0.041 0.68	-0.034 0.73	0.143 0.14
Stromal CD4+ TILs	-0.211 **0.03**	-0.198 **0.04**	-0.030 0.76	0.373 **< 0.0001**
Tumoral CD4+ TILs	-0.101 0.30	-0.024 0.80	0.058 0.56	0.256 **0.008**
Stromal FOXP3+ TILs	0.072 0.46	-0.066 0.51	-0.016 0.87	-0.23 0.81
Tumoral FOXP3+ TILs	0.068 0.49	-0.014 0.88	0.010 0.92	-0.81 0.41
Stromal CD20+ B-cells	-0.216 **0.02**	-0.232 **0.02**	-0.083 0.41	0.193 **0.04**
Tumoral CD20+ B-cells	-0.179 0.07	-0.189 0.06	-0.022 0.83	0.272 **0.005**
Stromal CD68+ TIMs	-0.145 0.14	-0.117 0.24	-0.054 0.59	0.326 **0.001**
Tumoral CD68^+^ TIMs	-0.062 0.53	0.015 0.88	0.071 0.47	0.022 0.82
Stromal CD163+ TIMs	-0.165 0.09	-0.167 0.09	-0.076 0.44	0.316 **0.001**
Tumoral CD163+ TIMs	0.033 -0.73	-0.020 0.84	-0.12 0.90	0.075 0.445

TILs, Tumor-infiltrating lymphocytes; TIMs, Tumor-infiltrating macrophages. The numbers of infiltrating CD8+, CD4+, and FOXP3+ T-cell lymphocytes, CD20+ B-cell lymphocytes, and CD68+ and CD163+ macrophages were evaluated by immunohistochemistry in both the tumor nests and surrounding stroma, and the mean numbers were correlated with the four systemic inflammatory ratios. The Pearson’s coefficients and the corresponding *p* values are shown.

## Discussion

In the present study we investigated the clinical relevance of four preoperative systemic inflammatory markers and their potential prognostic significance in OSCC. According to our results, NLR, SII and LMR showed prognostic value in the univariate analysis; however, only NLR remained an independent prognostic biomarker in the multivariate analysis. Recent studies have demonstrated that NLR may comprehensively reflect the inflammation and immune status of cancer patients (26), thereby representing a balance between the protumor inflammation and the anti-tumor immune status (27). The NLR indicates relative neutrophilia and lymphopenia, which has been related to shorter survival rates (11–13). Neutrophilia is known to inhibit the cytolytic activity of T- and NK cells (12), while a lymphopenia might underlie an insufficient host immune response due to destruction of lymphocytes by cancer cells (28). A recent meta-analysis conducted by Kumarasamy et al. (10) showed an overall positive correlation between increased NLR levels and a poorer prognosis in all but one included studies. In another meta-analysis, Takenaka et al. (11) found that two-thirds of studies demonstrated that a higher NLR was significantly associated with poor prognosis, whereas others led to non-significant results. Additionally, Ye et al. (29) failed to find any association between an elevated pre-treatment NLR and HNSCC patient survival, whereas Templeton et al. (15) reported that the prognostic impact of NLR might depend on the tumor site. Concordantly, using a multivariate analysis and adjusted HR we found that patients with a NLR value higher than 4.08 presented an enhanced risk of overall mortality. By contrast, SII, PLR and LMR had no influence on mortality risk in the multivariate Cox regression analysis. According to these results, we could hypothesize that the impact of these inflammatory markers on patient prognosis could plausibly be related to a surrogate role for poor performance status, advanced disease stage, and various comorbidities like cardiovascular disease and malnutrition. In fact, all these conditions affect OS but not DSS, as observed for the inflammatory markers. Malnutrition itself is related to a higher rate of postsurgical complications, higher tumor recurrence, increased risk of infections and poor prognosis (30). Moreover, various biochemical parameters, such as albumin or transferrin, but also blood cells like lymphocytes are markers commonly used in nutritional evaluation (31). The PLR, in turn, can be considered as a balance between the status of tumor growth and tumor suppression (32). It has been shown that elevated platelet counts are associated with an increased cancer incidence (33), hence suggesting that platelets could be involved in both carcinogenesis and tumor progression. The PLR has been used in predicting survival outcome, but according to the most recent studies, there is some controversy regarding the correlations between PLR and prognosis in cancer patients. Thus, the reported impact of thrombocytosis and PLR in HNSCC differs significantly among studies (34). In some reports, the PLR was associated with a better survival in multivariate analysis (35, 36), whereas PLR was not an independent prognostic biomarker in laryngeal cancer (37). As an extension of these data, in this study we did not find a relationship between the PLR and OSCC prognosis. High preoperative SII, which is related to high levels of circulating tumor cells (CTCs) (38), has been significantly associated with tumor size and advanced pathological grade in OSCC (22). The NLR was also associated with tumor size, while no significant associations were found between PLR and clinicopathological parameters. Also, patients with a high SII (above a cutoff value of 484.5) consistently showed a significant lower OS and DSS (22).

In the literature, low LMR has been associated with a poor prognosis in numerous solid cancers. Tham et al. (39) conducted a meta-analysis that included 4,260 patients with HNSCC in seven cohorts and found that an elevated LMR was significantly associated with improved OS. Higher amounts of lymphocytes in the TME have been correlated with improved prognosis (40), and conversely, macrophages derived from monocytes have been associated with poorer survival outcomes (41). Here we found that LMR calculated in peripheral blood specifically and significantly correlated with stromal infiltration of CD68+ and CD163+ macrophages but not tumor tissue infiltration. Macrophages represent an interface between innate and acquired immunity, and can be polarized into two phenotypes, proinflammatory and antitumor M1 and M2, which in turn increases inflammation and promotes tumor progression, immunosuppression, angiogenesis, migration, and metastasis (25). The prognostic role of tumor-associated macrophages (TAM) in tumors remains to be fully elucidated. Ohri et al. (42) reported that the number of M1 macrophages in the tumor tissue is associated with a better prognosis, whereas high infiltration of M2 macrophages in tumor tissue and stroma is associated with unfavorable prognosis (43). The mechanism by which M2 macrophages could be associated with a poor prognosis is strongly supported by their role in the promotion of tumor progression. Thus, M2 macrophages may facilitate PD-L1 expression by tumor cells (44), and the interaction between PD-1 and PD-L1 promotes immune suppression (45). Moreover, it has been recently demonstrated that exosomes derived from M2 TAMs promote cell viability, migration, and invasion in non-small-cell lung cancer (46).

CD68 is a pan-macrophage marker that allows to identify all macrophages regardless of their phenotype, while the CD163 is a marker for M2 macrophages. It has been reported that a high number of CD163+macrophages was significantly correlated with a worse survival in OSCC. Various studies showed that only stromal CD163+ expression resulted to be significantly associated with survival, whereas intratumoral CD163+ expression was not significant (47–49). As far as we know, this study is the first to assess and demonstrate the correlations between systemic inflammatory biomarkers and the local immune TME in OSCC tissue samples. Thus, NLR and SII were negatively correlated with stromal CD8^+^, CD4^+^, and CD20^+^ TIL density in the local TME. In addition, LMR was positively correlated with stromal infiltration of CD8^+^ TILs, tumoral and stromal infiltration of CD4^+^ and CD20^+^ TILs, as well as with stromal CD68^+^ and CD163^+^ macrophage densities in the local OSCC TME. CD8^+^ T lymphocytes identify and kill tumor cells playing a role in the anticancer immune response (50), and are usually supported by CD4^+^ T helper cells that release interferon-gamma (IFNγ) and interleukin-2 (IL-2) (51). Cancer cells express tumor-specific antigens that usually elicit an immune response mediated by CD20^+^ B cells, both as humoral immunity and modulating T cell responses to antigens (52). Consequently, neutrophilia and/or lymphopenia are correlated with the reduction of CD4^+^ T helper cells and cytotoxic CD8^+^ T cells in the tumoral stroma. Moreover, tumor-associated macrophages (TAMs) can be polarized into antitumoral M1 macrophages, expressing CD68, and protumoral and immunosuppressive M2 macrophages, characterized by coexpression of CD68 and CD163 (53). NLR, PLR and SII showed negative correlation coefficients with the different immune cell densities in the local OSCC TME. This could be due to systemic inflammatory response that leads to an increase in circulating neutrophils and platelets thereby reducing the number of lymphocytes available to act against tumor cells (54).

The mechanism behind the association between a high NLR, PLR, LMR or SII and poor cancer prognosis is not well understood (15). It is well known that cancer progression requires interactions between tumor cells and their microenvironment, including inflammatory, immune, and metabolic responses. It is therefore conceivable that immune cells execute their diverse functions both locally and systemically, boosting cancer initiation and progression (22).

Monocytes and lymphocytes have anti-tumoral effects (37). In particular, T cells are critical in the immune surveillance of cancer cells, and are involved not only in cytotoxic cell death, but also in cytokine production, which inhibits tumor cell proliferation and metastasis (55). In this regard, decreased lymphocyte density in tumor tissue has been associated with a poor outcome in OSCC (56). Cancer-mediated myelopoiesis has been related to the promotion of tumor angiogenesis, cell invasion and metastasis (38), and with persistence of immature myeloid cells (57). On the other hand, tumor cells can attract neutrophils that contribute to the destruction of the basement membranes and the invasion into surrounding tissues (58) through the secretion of epidermal growth factor (EGF), vascular endothelial growth factor (VEGF), interleukins IL-6 and IL-8 to promote tumor growth, invasion and metastasis (7).

However, neutrophils are also necessary for the recruitment of T cells that are associated with cytotoxic effects on tumor cells (13), In addition, neutrophils may release nitric oxide, arginase, and reactive oxygen species leading to T cell activation disorders (38). Neutrophils have been shown to contribute to tumor angiogenesis, mutagenesis, immunosuppression, and promotion of tumor cell proliferation (29, 59). Platelets promote tumor cell growth, epithelial-mesenchymal transition through secretion of TGF-β, vascular invasion, hematogenous dissemination, tumor cell extravasation (60), immune system evasion and the establishment of a metastatic niche (28), and even modulate the immune response (33). Neutrophil counts in the peripheral blood are elevated in OSCC patients (61). Tumor cells can increase the number of platelets in blood *via* thrombopoietin, IL-6 or leukemia inhibitory factor (62), and in turn, platelets facilitate tumor cell survival and escape from immune clearance mechanisms (63). Moreover, platelets could secrete transforming growth factor-beta (TGF-β) to suppress NK cells, and VEGF to promote tumor angiogenesis, ultimately contributing to tumor progression and metastasis (1, 37). In this study, our data reveal a relationship between NLR and SII with tumor size and clinical stage, which is in line with the aforementioned promotion of tumor growth and spreading. High NLR and SII might result from neutrophilia, thrombocythemia and lymphopenia, reflecting a combination of inflammation and dysfunctional immune system.

Interestingly, our study unveils high NLR as an independent prognostic factor for OS in OSCC, being superior to the other studied markers in predicting clinical outcomes.

In relation to the cutoff values for the different prognostic markers, they varied widely and were inconsistent among different individual studies. Hence, to define the optimal values for each cancer type might be a prerequisite for biomarker translation into clinical practice (22). In order to translate a continuous variable such as NLR, PLR, LMR or SII into a clinical decision tool, it is necessary to determine a cut-off point to stratify patients into distinct groups (64), optimizing the correlation with clinical outcomes by using minimization of p values or maximization of sensitivity and specificity tests. The majority of authors use a ROC analysis to determine optimal cutoff points; however, studies also often use cutoff values based on tertiles (65), quartiles (66), the median (67), or values referred to previous literature of different solid organs (68, 69). Herein, we were unable to determine reliable cut-off values based on ROC analysis to correlate NLR, PLR, LMR or SII with OS nor DSS. Thus, we based our analyses in percentiles and found that the percentile 90 was able to define statistically relevant prognostic groups for NLR and SII, and percentile 70 for the LMR. In a meta-analysis enrolling 3,770 patients from 16 studies that reported HRs for OS, Takenaka et al. (11) found that a higher NLR was associated with worse OS (HR =1.69) in 12 studies, whereas non- significant results were observed in the four remaining studies reported. These authors also found that the prognostic magnitude of NLR on OS was different depending on the tumor sites, with a highest value for oropharyngeal cancer (HR = 4.60) and lower for OSCC (HR = 1.50). Mascarella et al. (13) conducted a meta-analysis and found a combined HR for OS of HNSCC patients and an elevated NLR of 1.78, and NLR of 1.56 for OSCC. Yu et al. (14) performed another recent meta-analysis that included 5,475 patients found a HR of 1.84 for OS in patients with a high NLR value. The HR found in the present study was 1.81. It is important to note that NLR may also be elevated in benign disorders such as renal and coronary heart disease (70, 71). The reported NLR cutoff points used for OS in HNSCC patients ranged from 1.92 to 5.56 (12, 13); the PLR cutoff varied in a range of 105.3 to 170 (72–74) including several head and neck tumor locations; and the published LMR cutoff values range from 2.475 to 5.3 (75). The cutoff values used in this study for NLR, PLR, and LMR were 4.08, 162, and 4.58, respectively, values that are within the published ranges, taking into account that threshold values and clinical significance differed significantly by primary sites (76). It is plausible that cutoff values could vary depending on the genetic background for different tumor cell origin, etiological factors or even for different patient populations and ethnicities (39). In fact, the majority of the studies have been undertaken in East Asian populations (39, 77). Yu et al. (14) inferred that the prognostic value of NLR in HNSCC patients is influenced by the cutoff and recommended to use a continuous range of NLR values, rather than point values. Furthermore, OSCC patients frequently present with ulcerative and infected lesions that lead to an increased neutrophil count. Consequently, the most discriminatory cutoff points for systemic inflammatory markers remain unknown and may differ among different tumor sites and stages.

There are some limitations of this study. This study is retrospective in nature, and there may be selection bias during retrospective data collection. Additionally, our information on hematological markers only reflect the preoperative status, and it is conceivable that post-treatment markers could change and predict clinical outcomes. Moreover, subsites, stages, treatment modalities, cutoff methods, cutoff values, and data analysis were quite different among individual studies. Furthermore, OSCC is often ulcerated, and heavily infiltrated by immune and inflammatory cells when exposed to the microbial environment of the oral cavity. Hence, they could be confounding factors when comparing ulcerated and non-ulcerated tumors in relation to the immune tumor microenvironment.

## Conclusion

In conclusion, our results reveal that preoperative determination of the systemic NLR, SII, and LMR may serve as easily and valuable markers to predict OSCC patient prognosis, together with the TNM stage and the patients’ age. Moreover, high NLR was found an independent prognostic indicator of poor OS. Notably, these systemic inflammatory markers were well correlated with the local immune cell densities in the stroma of tumor microenvironment. Further prospective studies are warranted to clarify and validate the prognostic value of NLR and the accurate cutoff values before translation into daily clinical practice.

## Data availability statement

The raw data supporting the conclusions of this article will be made available by the authors, without undue reservation.

## Ethics statement

The studies involving human participants were reviewed and approved by Regional Ethics Committee from Principado de Asturias (date of approval 14th of May 2019; approval number: 136/19, for the project PI19/01255). The patients/participants provided their written informed consent to participate in this study.

## Author contributions

All authors contributed to the study conception and design. Material preparation, data collection, and analysis were performed by MR-R, PL-F, TR-S, FS-S, and RL-P. JMG-P contributed to the design of the study, data interpretation and review of the manuscript. JCV contributed to the design of the study, data analysis and interpretation, and wrote the first draft of the manuscript. All authors read and gave final approval of the final version of the manuscript.

## Funding

This study has been funded by Instituto de Salud Carlos III (ISCIII) through the projects “PI19/01255” and “PI19/00560” and co-funded by the European Union, and also the Instituto de Investigación Sanitaria del Principado de Asturias (ISPA), Fundación Bancaria Caja de Ahorros de Asturias-IUOPA, and the FEDER Funding Program from theEuropean Union.

## Conflict of interest

The authors declare that the research was conducted in the absence of any commercial or financial relationships that could be construed as a potential conflict of interest.

## Publisher’s note

All claims expressed in this article are solely those of the authors and do not necessarily represent those of their affiliated organizations, or those of the publisher, the editors and the reviewers. Any product that may be evaluated in this article, or claim that may be made by its manufacturer, is not guaranteed or endorsed by the publisher.

## Supplementary material

The Supplementary Material for this article can be found online at: https://www.frontiersin.org/articles/10.3389/fimmu.2022.941351/full #supplementary-material
