# Supplementary material for: Prognostic implications of preoperative systemic inflammatory markers in oral squamous cell carcinoma, and correlations with the local immune tumor microenvironment
Source: Front Immunol. 2022 Jul 26;13:941351. doi: 10.3389/fimmu.2022.941351 (PMC9360320; doi:10.3389/fimmu.2022.941351)
Supplement: Supplementary file 1 [file Table_1.docx]

**SUPPLEMENTARY INFORMATION**

**Supplementary Table S1. Univariate Cox regression analysis of overall and disease-specific survival in the subgroup of 184 OSCC patients who received postoperative radiotherapy.**

| **Variable** | **OS** | | **DSS** | |
| --- | --- | --- | --- | --- |
|  | **HR (95% CI)** | ***p*** | **HR (95% CI)** | ***p*** |
| **Age (years)**  **< 65**  **≥ 65** | 1 (reference)  1.676 (1.187 – 2.366) | 0.003 | 1 (reference)  1.662 (1.142 – 2.420) | 0.008 |
| **Gender**  **Female**  **Male** | 1 (reference)  0.983 (0.685 – 1.411) | 0.92 | 1 (reference)  0.818 (0.556 – 1.204) | 0.30 |
| **Tobacco**  **No**  **Yes** | 1 (reference)  0.755 (0.517 – 1.102) | 0.14 | 1 (reference)  0.643 (0.431 – 0.960) | 0.03 |
| **Alcohol**  **No**  **Yes** | 1 (reference)  0.837 (0.600 – 1.166) | 0.29 | 1 (reference)  0.702 (0.488 – 1.010) | 0.05 |
| **AJCC pT**  **pT1**  **pT2**  **pT3**  **pT4** | 1 (reference)  0.641 (0.401 – 1.022)  1.171 (0.669 – 2.051)  1.518 (0.918 – 2.511) | 0.001  0.06  0.58  0.104 | 1 (reference)  0.698 (0.411 – 1.185)  1.281 (0.679 – 2.416)  1.963 (1.137 – 3.390) | <0.0001  0.18  0.44  0.01 |
| **AJCC pN**  **pN0**  **pN+** | 1 (reference)  1.259 (0.881 – 1.801) | 0.20 | 1 (reference)  1.255 (0.847 – 1.860) | 0.25 |
| **Stage**  **I**  **II**  **III**  **IV** | 1 (reference)  0.664 (0.356 – 1.241)  0.882 (0.498 – 1.562)  1.277 (0.766 – 2.128) | 0.05  0.10  0.66  0.34 | 1 (reference)  0.943 (0.464 – 1.914)  1.095 (0.557 – 2.151)  1.680 (0.919 – 3.071) | 0.06  0.87  0.79  0.09 |
| **Grade**  **Well**  **Moderate + Poor** | 1 (reference)  0.973 (0.692 – 1.367) | 0.87 | 1 (reference)  0.993 (0.683 – 1.444) | 0.97 |
| **NLR**  **≤ 4.08**  **> 4.08** | 1 (reference)  1.418 (0.793 – 2.534) | 0.23 | 1 (reference)  1.142 (0.592 – 2.205) | 0.69 |
| **PLR**  **≤ 205**  **> 205** | 1 (reference)  1.373 (0.734 – 2.565) | 0.32 | 1 (reference)  1.173 (0.588 – 2.341) | 0.65 |
| **SII**  **≤ 1,137**  **> 1,137** | 1 (reference)  1.556 (0.848 – 2.853) | 0.15 | 1 (reference)  0.883 (0.407 – 1.916) | 0.75 |
| **LMR**  **≤ 4.58**  **> 4.58** | 1 (reference)  0.720 (0.471 – 1.103) | 0.13 | 1 (reference)  0.636 (0.387 – 1.044) | 0.07 |
